# Religiosity of Latinas Living in the USA Curbs Depression and Anxiety During the COVID-19 Pandemic

**DOI:** 10.1007/s10943-024-02038-z

**Published:** 2024-04-13

**Authors:** Maud Joachim-Célestin, Nishita Matangi, Jemima Ruth Bagcus, Susanne B. Montgomery

**Affiliations:** 1 Department of Interdisciplinary Studies, Loma Linda University School of Behavioral Health, 11155 Mountain View Ave., Suite 226, Loma Linda, CA 92354 USA; 2 Department of Preventive Medicine, Loma Linda University School of Medicine, 11175 Campus Street, Loma Linda, CA 92350 USA; 3 Department of Social Work, Loma Linda University, 1898 Business Center Drive, San Bernardino, CA 92408 USA; 4 Rosemead School of Psychology, Biola University, 13800 Biola Avenue, La Mirada, CA 90639 USA; 5 School of Behavioral Health, Loma Linda University, Griggs Hall 224, 11065 Campus St., Loma Linda, CA 92350 USA

**Keywords:** Religiosity, COVID-19 pandemic, Latina, Mental health

## Abstract

This mixed-methods study was conducted to explore the role of faith in mental health among Latino women (Latinas) during the COVID-19 pandemic. As part of a lifestyle study, surveys were administered to 89 participants during the 1st year of the pandemic. Specifically, a focus group was conducted with participants (*n* = 6) directly affected by COVID-19 (i.e., self or family member). The results showed inverse correlations between religiosity and both depression and anxiety, as well as positive correlations among religious practices, religious coping, and religiosity. Given these associations, future interventions should explore the role of faith in supporting individuals during difficult times.

## Introduction

The COVID-19 pandemic caused much uncertainty and fear, increasing stress, anxiety, and depression among both health professionals and the general population (Şimşir et al., 2022). This was especially true among Hispanic/Latinx populations, which are often more impacted by social determinants of health, such as food and housing insecurity, as evidenced by 47% of Latinx households with children experiencing food insecurity in 2020, a stark contrast with 16.8% of Latinx households with children experiencing food insecurity prior to the pandemic (Schanzenbach & Pitts, 2020). For Latino women (Latinas), especially mothers, housing conditions and the burden to continue caring for their families as caregivers made it difficult for them to isolate even if they were infected (Sehgal et al., 2021).

Moreover, higher rates of job, housing, and food insecurity among Hispanic/Latinx populations, in conjunction with disparities in exposure, cases, and deaths, resulted in much higher rates of mental health issues in these groups. In the USA, 28.6% of Hispanic individuals experienced depression, 18.2% initiated or increased their substance use, and 8.4% experienced suicidal thoughts during the peak of the pandemic. Indeed, Hispanic adults reported having more symptoms of depression (59% more symptoms than White adults), and suicidal ideation was four times more common among Hispanic individuals than among Black or White individuals (McKnight-Eily et al., 2021).

In times of uncertainty or natural disasters, the role of religiosity has been explored as a moderator of physical and mental health. When people have less control over their lives and face uncertainty, they often turn to religion as a coping strategy (Almaraz et al., 2021; Pirutinsky et al., 2021; Stege & Godinez, 2022). Religiosity has been defined as “an organized set of beliefs and practices that involves various dimensions of one’s cognitive, behavioral, and sociocultural well-being across one’s lifespan” (Bergan & McConatha, 2001). Somewhat related is the concept of religious coping, defined as a set of beliefs, religious activities, and social support systems that are utilized in the face of adversity and often contribute to mental health and well-being (Pirutinsky et al., 2021).

Expanding the range of terms, Pargament et al. (1998) distinguished two types of religious coping: positive and negative religious coping. Supporting studies have referred to positive religious coping as a secure relational attachment to God, spiritual connectedness with others, and belief in life’s meaning and purpose (Stege & Godinez, 2022). On the other hand, negative religious coping is associated with the idea of punishment and abandonment by God, and adversity is considered the consequence of sinful behavior (Herrera et al., 2009).

While some studies suggest religiosity as a means of reducing psychological distress and promoting well-being, others have highlighted the harm that religiosity may cause individuals with negative religious beliefs and experiences (Herrera et al., 2009; Pargament et al., 2001; Stege & Godinez, 2022). Recently, religious coping has been a focal point of study in the context of the COVID-19 pandemic, and the results have been mixed.

Almaraz et al. (2021) assessed the role of trust and mistrust in God and found negative correlations between mistrust in God and positive emotions as well as social support. The authors of a study conducted in an American Jewish community concluded that higher intrinsic religiosity and positive religious coping were correlated with less negative impacts of the COVID-19 pandemic on one’s emotions (Pirutinsky et al., 2021). Among Indonesian Muslims residing in Indonesia, religion and religious coping reduce anxiety and promote well-being (Achour et al., 2021; Saud et al., 2021).

A study conducted among Seventh-day Adventists in Germany suggested a positive correlation between spiritual well-being and religious practices (a high frequency of prayer and meditation) and emotional well-being during the pandemic. This association was mediated by gratitude. However, among members not in leadership roles, spirituality was strongly correlated with fear of the future (Büssing et al., 2022). Another study, this time among Muslims living in Pakistan, reported a positive correlation between religious coping and anxiety (Mahmood et al., 2021).

In the USA, approximately 80 to 95% of Mexican-origin Latinx people report having a religious affiliation or participating in religious activities, which reflects the strong religious values within Latinx culture. These characteristics are more common among Mexicans born in Mexico than among US-born Mexicans (Noyola et al., 2020). Recent studies have confirmed the prominence of religious and spiritual practices as well as positive regard for prayer, church attendance, and personal trust in God among Mexican individuals. The impact on study participants’ mental health was also reported (Stege & Godinez, 2022).

Given the influential relationship between religiosity and mental health, as well as the unique cultural values upheld by the Latinx population, this mixed-methods study sought to explore the relationship—if any—between religiosity and mental health among Latinas living in southern California during the COVID-19 pandemic. To the authors’ knowledge, this is the first study to explore religiosity among Latinas during the COVID-19 pandemic.

## Methods

### Data Collection

This mixed-methods study was designed as a pilot study (substudy) within a larger study (a lifestyle intervention study) and included 2 quantitative data collection points—one 7 months into the larger study, and the other at approximately the one-year mark—points that approximately coincided with the 7th and the 12th months of the COVID-19 pandemic. Both data collection points (for this substudy, 7 months was considered the baseline and 1 year was considered the second collection point) immediately followed periods of regional peak pandemic spikes. The purpose of this substudy was to evaluate the mental health changes experienced by some of our respondents.

Participants (*n* = 89) were Latinas aged 18 years or older who were enrolled in a culturally adapted lifestyle intervention for overweight and obese Latinas provided in the Inland Empire (southern California)—described elsewhere (Joachim-Célestin et al., 2022)—and who responded to an additional short survey regarding COVID-19 during the pandemic.

Written and verbal informed consent was obtained from each participant prior to data collection, and each participant received a US $10 certificate to a local grocery store at each data collection point. This study was performed in accordance with the principles of the Declaration of Helsinki*,* and ethical approval for the study was obtained from the Loma Linda University Institutional Review Board (IRB #518,068).

The quantitative data, including demographic data (i.e., acculturation level and religiosity status) collected prior to the pandemic, were self-reported. Food insecurity, depression, and anxiety were assessed at 7 and 12 months after the start of the pandemic. Questions assessing housing status and household members’ job settings, gratitude, and religious practices used to cope with the pandemic were also included in the surveys at these times.

Because the variables did not satisfy all the assumptions for normality, we performed sample size analyses for Spearman’s rank correlations. Spearman’s rank correlation coefficient is the same (computationally) as the Pearson product–moment coefficient, so we estimated the power of Pearson’s correlation. We used an alpha of 0.05, a power of 0.80, and a medium effect size of 0.3 for a two-tailed test. Based on the above assumptions, the required sample size was determined to be 84.

Qualitative data were collected in early October 2020, approximately 6 months after the initial COVID-19 lockdown, through a focus group discussion (FGD) with select participants (*n* = 6). A researcher and a community health worker facilitated the FGD. Focus group participants who had been directly affected by COVID-19 (i.e., being infected themselves or having infected family members within the same household) were selected. The FGD was conducted via Zoom after the participants answered a short seven-question survey assessing housing, job, and food insecurity. The recording from the FGD was transcribed, translated by a bilingual researcher and then analyzed for themes using NVIVO software.

### Measures

The quantitative measures of intrinsic religiosity, positive religious coping, religious practices, gratitude, depression and anxiety, and acculturation are described below.

#### Intrinsic Religiosity

Intrinsic religiosity was measured using the statement “My religious beliefs are what truly lie behind my whole approach to life,” an item from the Duke University Religion Index (DUREL) that is predictive of intrinsic religiosity (Koenig & Al Zaben, 2021; Koenig & Büssing, 2010). Response options were based on a 4-point Likert scale ranging from “0” (“definitely not true”) to “4” (“definitely true”).

#### Positive Religious Coping

Positive coping was measured using four items from the validated measure of religious coping with major life stressors (RCOPE)—short form: “looked to God for strength,” “support and guidance,” “sought God’s love and care,” and “trusted that God would be by my side” (Martinez & Sousa, 2011; Pargament et al., 1998, 2000). Response options were based on a 4-point Likert scale ranging from “0” (“definitely not true”) to “4” (“definitely true”). Responses were then averaged for each participant to determine the positive religious coping index. The Cronbach’s alpha was 0.939.

#### Religious Practices

The religious practices variable was a new proxy variable created by combining the responses to the question answered at the end of the program: “What other things are you doing to stay healthy during this time? Check all that apply.” The response options included “meditation,” “worship/church attendance” (online or in person), and “prayer” and were selected from the validated SpREUK questionnaire on religious practices (Büssing et al., 2005). One point was assigned for each item checked, and the number of points was added to create this ordinal variable, allowing for bivariate correlations. The answers ranged from 0 (none of the suggested practices were selected) to 3 (all 3 religious practices were selected).

#### Gratitude

Gratitude was assessed based on the response to the following question: “What are some positive things that you are thankful for at this time? Mark all that apply.” Each checked answer was given 1 point, and the number of points was added to create an additional variable (“number of expressed reasons for gratitude”).

#### Depression

The Patient Health Questionnaire-9–Spanish Version (PHQ-9–Spanish) developed by Zhong et al. (2014), which originated from Spitzer et al. (1999) original PHQ-9, was used to assess depression. The internal consistency was moderate (PSI = 0.72). Response options were based on a 4-point Likert scale ranging from “0” (“not at all”) to “3” (“nearly every day”).

#### Anxiety

The Generalized Anxiety Disorder-7 (GAD-7) is a measure that was created to identify probable cases and the severity of symptoms of generalized anxiety disorder (GAD) and has been validated among Spanish-speaking Latinx individuals (García-Campayo et al., 2010; Spitzer et al., 2006). This 7-item anxiety questionnaire was based on a general anxiety diagnosis according to the DSM-IV criteria as well as 4 items developed using existing anxiety scales. Response options were based on a 4-point Likert scale ranging from “0” (“not at all”) to “3” (“nearly every day”).

#### Acculturation

The acculturation level was based on the answers to the validated Brief Acculturation Scale for Hispanics, which determines acculturation according to personal and household language preferences (Mainous et al., 2008).

## Results

### Quantitative Results

Table 1 shows the sociodemographic characteristics of the study participants at baseline and at one year (the end of the study). The participants in this study were mostly low-income Latinas—a population at greater risk for COVID-19 infection—with the majority (79.8%) being married, and born in Mexico (80.9%); almost all (96.2%) had lived in the USA for more than 10 years yet had a low acculturation level. At baseline, most (84.3%) of the participants lived in a household of 3 or more individuals, 92.1% had more than one child, and almost all (97.7%) were primary caretakers who cooked most—if not all—meals for their households.

**Table 1:** Sociodemographic characteristics of the study participants

Variables		Study baseline (7 months)	End of study (12 months/end of the year)
		*n* (%)/mean (SD)	*n* (%)/mean (SD)
Age		50.72 (12.32)	49.68 (10.52)
Marital status	Single never married	5 (5.6)	4 (5.9)
	Married	71 (79.8)	55 (80.9)
	Divorced or separated	16 (12.3)	8 (11.8)
	Widowed	2 (2.2)	1 (1.5)
Number of household members	2 or less	14 (15.7)	8 (10.3)
	3 or 4	38 (42.7)	24 (35.3)
	5 or more	37(41.6)	36 (52.9)
Religious affiliation	Christian (Protestant)	67 (79.8)	67 (79.8)
	Christian (Catholic)	17 (20.2)	17 (20.2)
Number of children	0	3 (3.4)	1 (1.5)
	1	4 (4.5)	4 (5.9)
	2 to 4	71 (79.8)	56 (82.3)
	5 or more	11 (12.4)	7 (10.3)
Birth country	USA	10 (11.2)	1 (1.5)
	Mexico	72 (80.9)	63 (92.6)
	Central/South America	7 (7.8)	4 (5.9)
Years lived in the USA	1 to 10 years	3 (3.8)	4 (6.3)
	11 to 20 years	21 (26.6)	29.1 (34.3)
	> 21 years	55 (69.6)	38 (59.4)
Family income	< $21,000	29 (42.6)	9 (16.4)
	$21—$50,999	31 (45.6)	38 (69.1)
	$51—$75,000	5 (7.4)	7 (12.7)
	> 75,000	3 (4.4)	1 (1.8)
Food insecurity	Not food insecure	55 (63.2)	29 (43.9)
	Food insecure	32 (36.8)	37 (56.1)
Employment	Unemployed	63 (72.4)	50 (73.5)
	Employed	24 (27.6)	18 (26.5)
Who cooks most	Cooks most of the time	87 (97.7)	65 (97)
	Doesn’t cook most of the time	2 (2.3)	2 (3)

Additionally, 63% of the participants were unemployed, 85.5% had a family income of less than $51,000, and 36.8% were food insecure. Although not shown in Table 1, approximately half (45%) of the participants had less than a high school diploma, and only 8.7% had received a bachelor’s degree. The participants who reported religiosity were Christians of either Catholic (20.2%) or Protestant (79.8%) faith.

Notably, while we experienced participant loss over time, there were only modest differences in the participant demographic profiles between the baseline (7 months) and one-year data collection points. Differences included greater food insecurity at one year, and slightly lower incomes at baseline. For context, by the 7th month of the pandemic, when asked about family income and whether a household member was an “essential worker” (i.e., someone who worked outside of the home regardless of the number of COVID-19 cases in the work area), 85% of the participants reported having less family income during the pandemic than before the pandemic, and 86% had an “essential worker” living in their household. Table 2 shows the correlations between religiosity and mental health.

**Table 2:** Correlations among religiosity, mental health, and gratitude during the pandemic

	1	2	3	4	5	6	Religious practices
1. Positive religious coping	–						0.20*
2. Intrinsic religiosity	0.80*	–					0.19*
3. Anxiety at 7 months	− 0.20	− 32*	–				− 0.12
4. Anxiety at the end of the program	− 0.29*	− 0.30*	0.73*	–			− 0.14
5. Depression at 7 months	− 0.18	− 0.31*	0.77*	–	–		− 0.04
6. Depression at the end of the program	− 0.20	− 0.25*	0.69*	–	–	–	− 0.16
Number of items grateful for	0.09	0.06					0.45*
Grateful for family time	0.07	0.06					0.37*
Grateful for health	0.008	− .04					0.29*
Grateful for no exposure	0.10	0.03					0.17*
Grateful for physical activity	0.07	0.12					0.19*
Grateful for the ability to prepare and eat healthy foods	0.07	0.12					0.27*
Grateful that the world united	0.09	0.08					0.36*
Grateful for sleep	0.05	− 0.03					− 0.15
Grateful for extracurricular activities	0.05	0.006					0.25*

^*^Correlations were considered significant at the 0.05 level (two-tailed)

Participants who had high scores for positive religious coping also had high scores for intrinsic religiosity (*r*_s_ = 0.80, *p* < 0.001). They were also less likely to report anxiety at the end of the program (*r*_s_ = −0.29,* p* = 0.02), and intrinsic religiosity was inversely correlated with depression and anxiety at 7 months (*r*_s_ = −0.31, *p* = 0.02 and *r*_s_ = −0.32, *p* = 0.01, respectively) and 12 months (*r*_s_ = −0.25, *p* = 0.04, *r*_s_ = −0.30, *p* = 0.02, respectively) after the start of the pandemic. Intrinsic religiosity and positive religious coping were positively correlated with the number of religious practices (*r*_s_ = 0.19, *p* = 0.04 and *r*_s_ = 0.20, *p* = 0.03, respectively), and the latter was also positively correlated with the number of items the participants were grateful for (*r*_s_ = 0.45, *p* < 0.001). This was especially true regarding gratitude for family time and for “seeing the world unite” (*r*_s_ = 0.37 and *r*_s_ = 0.36, *p* < 0.001, respectively).

When performing post hoc power analyses for all nonstatistically significant correlations between the number of religious practices and gratitude for sleep, the results (power) ranged between 0.92 and 0.98.

### Qualitative Results

Based on the short surveys completed by the focus group participants, four participants lost their jobs, and five knew of someone who had lost their jobs due to the pandemic. One participant lost her home, and four participants feared that they were in danger of losing their homes. All but one participant experienced food insecurity.

When analyzing the qualitative data, five themes emerged: fear of self-/family contracting COVID-19; mental health symptoms; factors attributed to Latinx culture; faith in God; and gratitude. All the participants experienced worry and fear, which led to an increase in mental health challenges (depression and anxiety). However, faith and trust in God as a coping mechanism was a prominent, emergent theme. Gratitude for God, family, and support groups was also expressed and credited for the outcomes (see Table 3).

**Table 3:** Themes derived from focus group discussions

Themes	Quotes
1. Fear of self/family contracting COVID-19	
	*Yes, sorry doctor, you see, I was terrified of my kids being exposed* *The truth is I had a fear, and that would mess with me psychologically, especially because I was always worrying about where and when I was touching anything and how I had to immediately sanitize* *Yes, yes. With my parents, it was harder since these were some distressing moments because we didn't know what to expect. Would the medications we receive work?*
2. Mental health symptoms	
a. Depression	*I was depressed at that time. I was a little depressed because of all that worrying* *There was a lot of worrying and I was depressed, I didn't want to, there wasn't a moment where I wouldn't think “What will happen? What will happen now?” I was always asking that he wouldn’t die and well, in the end, it was honestly a very difficult situation*
b. Anxiety	*I don’t know if this has happened to the rest of the ladies but the symptoms and the anxiety has affected me; I never suffered with this before …*
3. Factors attributed to Latino culture	
a. Perceived lack of belief in COVID-19 among Latinos as a source of concern	*I also believe that as a culture we struggle with this because of a cultural stigma. I have had a lot of people, friends actually, that tell me that they believe they had it because they showed some symptoms. However, the truth was that they haven’t gotten tested. I don’t truly know where you can find people like this but I do think it’s kind of irresponsible on their parts* *I have also heard Latinos being incredulous because they say it doesn’t exist and that it’s just the flu and all that. So, in that way we are pretty irresponsible, maybe not everyone but the majority of people I know has that way of thinking*
b. Caretaker role of Latino women	*I was the one responsible for keeping everyone in check. In the evening I would be exhausted and have headaches* *And well, I wouldn't say anything and um, there was this one time I told my daughter, “If I’m positive and if I get bad with a virus,” and I say this because—well, sometimes they push you to prepare the meals and all that—“What are we going to do?” and “You guys are going to be in charge of the meals.” She told me “No mom, don’t worry, you keep preparing the meals because either way we are all going to be infected.” Hehe*
	*Yes, I continued to cook but I believe it does affect us because we love to always serve the family and it's something that might be dangerous as well because in this case when you are infected, you infect the family* *… Um I, I would help with my parents and stay the day with them and come back to my house to sleep. I would go straight to my room and try to isolate myself from everyone else; also with my kids. But I don’t remember thinking about eating healthy or taking some time for myself. That comes like a second thing and not a priority*
c. Strong social network as a “neutralizing” factor	*I also think that it is something cultural because, at least in my family, we would get together at least once a month or every Sunday after mass to visit the parents and things like that, ahh…* *However, on the question of what it has to do with the Latino community, I believe, just like someone else shared, we are motivated to stay together, to serve the family, to serve the kids*
d. Resiliency of the Latino community	*I think that my faith, our family bond, and I think that as a Latino community we are more resilient*
4. Faith in God	
	*Yes, I think it is my faith, my religion, my trust in God, and putting all I have in His hands even though it was a time of desperation that we lived but it was like, “we know that we are here today, but we don’t about tomorrow and leave it to God and His will…”* *I believe that a part of helping was also my trust in God, that if we were infected, we would be in His hands*
5. Gratitude	
a. Being grateful to God	*Yes, I did take the precautions and all of that but it’s thanks to God that today I still do my best to follow the safety precautions* *I have been worried and anxious lately but it’s all thanks to God that this didn’t affect me heavily…*
b. Being grateful for community/family	*Since he doesn’t have family close by and they are in Mexico. He talked to his family and everyone was always sending him messages and making video calls. He was always busy, haha. Yes, always on the phone and I think that helped him recover a lot…* *Another one was the connection I had with my kids. My kids, my husband, and I have been truly close these last 4 weeks* *Another thing I did, since I was isolated, I decided to follow two support groups, I told myself “I have the computer and my phone,” and I was able to connect with two groups that also focused on COVID-19 and people who are recovering from it and are also going through the same experience as me. That was a big help and it still is for me*

Overall, worry negatively affected the participants’ mental health. Every focus group participant admitted that the experience took a psychological toll on them: The process of attempting to prepare emotionally for whatever the pandemic would bring was associated with feelings of depression or at least a sense of distress. These women also shared their fears: fear of contracting the virus, fear of spreading the infection, and fear of the future (e.g., job insecurity, whether medicine/treatment would work for those who had COVID-19, fear of the health care system).

The following cultural factors that are specific to Latinas were also discussed: the incredulity of fellow Latinx individuals toward COVID-19 infection and the prominent role of women at home. The perception that other Latinx people did not take the pandemic seriously was particularly concerning. The participants felt that, once sick, fellow community members avoided being tested due to fear of being isolated as a result of hospitalization. There was also mistrust and misinformation in the Latinx community concerning the seriousness of the COVID-19 pandemic.

The cultural expectations of caregivers pressured the participants to fulfill the demands of their role even when they became sick. This role added stress and worry, thereby compounding their mental health challenges. Nevertheless, the participants reported growing closer to their family and rallying to encourage family members with COVID-19, supporting them whenever needed, and finding a way to be positive and to cope.

Participants attributed all behaviors and values to the resiliency of Latinx culture. The participants also attributed their ability to cope with the emotional struggles and fears they experienced when a household member contracted COVID-19 to their religious beliefs, such as trusting that God is a reliable source of strength. They credited God for bringing their family through the pandemic, for their ability to take necessary precautions and surmount obstacles, for sparing them from contracting the virus, and for their experience of relatively mild anxiety. The participants also expressed gratitude to God for social connections such as support groups and their church family, with whom they could spend time despite the pandemic.

## Discussion

The purpose of our study was to explore associations between religiosity and mental health in Latinas during the COVID-19 pandemic. Our results indicated that religiosity was beneficial to these women during at least the first 12 months of the pandemic, as it was associated with less depression and anxiety.

This study’s quantitative and qualitative findings reflect the challenges faced by Latinx women during the 1st year of the pandemic: housing, job, and food insecurity and mental health symptoms (Blanco et al., 2022; Ornelas et al., 2021). In the inland area, reported cases and deaths increased dramatically between the 7th and 12th months after the start of the pandemic, and in our study population, more than three-quarters of the participants reported a decreased family income despite most households having a family member working as an “essential worker.” Exposure to these types of insecurities is known to cause stress, which in turn affects mental health (Cleaveland & Frankenfeld, 2023).

Indeed, the novelty of the virus and the pandemic were correlated with poor mental health among Latinx individuals. Although the focus group participants believed that the Latinx culture conferred some benefits in that it encouraged them to rally together to support family members, it is obvious that these benefits were not enough to compensate for the challenges they and other Latinx individuals faced, as evidenced by the depression levels reported in this population during the 1st months of the pandemic.

Among Latinx individuals nationwide and in the study geographic area, depression and anxiety scores were extremely high among Latinx individuals during the 1st year of the pandemic, especially among those with less than a high school education, accounting for half of the women in our study (Cleaveland & Frankenfeld, 2023). Our study participants’ depression and anxiety scores were the highest at the one-year follow-up, although they expressed experiencing high levels of anxiety and depression 7 and 12 months after the start of the pandemic.

With the relatively high percentage of “essential workers” among our participants, it is understandable that there would be much concern and fear about either becoming infected with COVID-19 or spreading the virus. This fear was clearly expressed by our study participants during the focus group discussion.

One reason for the fears of the participants was the perceived laissez-faire attitude of fellow Latinx individuals, which they attributed to cultural beliefs—an attitude that increased their risk of contracting COVID-19. Another cause of fear was the pressure to remain in close proximity to others and provide care for family members, increasing the risk of infection for the participants and others interacting with them. Molina et al. (2019) also reported the unique distress that Latinas experienced as a result of familial obligations.

Having dealt with the infection first-hand may have played a role in the participants taking the disease more seriously, while others around them did not. It would have been interesting to see if there was a difference in fear levels between groups with different levels of religiosity, but this information was not available.

An increase in fear of the future was also reported by authors of a study among Seventh-day Adventist lay members (i.e., not church leaders) living in Germany (Büssing et al., 2022) during the pandemic. However, in that study, participants expressed fear of the future for other reasons: According to the authors, the fear was due to the perception that society was “falling apart” and was correlated with more intense prayer and meditation. Thus, fear seemed to be associated with higher religiosity. The authors proposed that this correlation may be the result of specific beliefs held among Seventh-day Adventists about the end of the world (Büssing et al., 2022).

In other studies conducted prior to the pandemic, religiosity, even among professed Christians, was associated with depression resulting from feelings of guilt or a sense of conflict as individuals faced mental health challenges (Bonelli et al., 2012; Mitchell & Romans, 2003). However, the present study suggested that positive religious coping among Latinas of Mexican descent with Christian beliefs may be beneficial. Within this sample, in particular, expressions of gratitude in conjunction with Latinas’ trust in God seemed very much related to religious practices and seemed to be effective positive religious coping mechanisms counteracting culturally amplified COVID-19 stressors.

Furthermore, religious practices have been shown to be positively correlated with gratitude to God, as reported by Büssing et al. (2021). In our study, the more the participants engaged in religious practices, the greater the number of items for which they expressed gratitude. However, gratitude was not significantly directly correlated with religiosity or positive religious coping. This may be due to the lack of specific measurements of gratitude.

There seemed to be a buffering effect of religiosity among individuals with higher intrinsic religiosity (when compared to those who had lower intrinsic religiosity) in that they reported less depression and anxiety symptoms at 7 months after the start of the pandemic and even at 12 months, immediately after a dramatic increase in the number of local cases and deaths from COVID-19 (California Department of Public Health, 2023). Furthermore, less anxiety was reported among individuals with positive religious coping skills. There was also a strong correlation between intrinsic religiosity and positive religious coping, and these factors were both positively associated with the number of religious practices.

The quantitative results were supported by our qualitative data: The participants’ ability to cope with the many stressors they faced during the pandemic was attributed to God and faith. Our results support previous research findings showing that higher religiosity was a protective factor of mental well-being during the pandemic. Among some Muslim groups, religiosity has been associated with less anxiety during the pandemic (Achour et al., 2021; Saud et al., 2021).

A similar phenomenon was found among individuals from a Jewish community where higher intrinsic religiosity was positively correlated with life enjoyment, while positive religious coping was inversely correlated with strong negative emotions (Pirutinsky et al., 2021). Stege and Godinez (2022) also found positive relationships between trusting in God for strength and a greater likelihood of engaging in religious coping strategies, associations supported by the results of our study.

Moreover, the qualitative themes identified in this study further supported the quantitative findings that religiosity is associated with less anxiety and depression. The extent to which the lifestyle intervention or social interaction between participants increased the impact of religiosity on mental health is unknown. However, the fact that depression and anxiety were not correlated with any of the religious variables prior to the pandemic or with attendance in the lifestyle intervention seems to indicate that the intervention did not influence these results.

One of the strengths of this study is that data were collected from a subset of individuals participating in a longitudinal pilot study of a lifestyle intervention during the worst part of the pandemic for Latinx individuals, allowing for the collection of rich data at two time points specific to a time of crisis. The mixed-methods study (i.e., the inclusion of qualitative responses) design allowed us to corroborate findings from the quantitative data with the nuanced and explorative details that came from the focus group discussion.

In addition, the qualitative approach gave us the opportunity to explore topics or concerns that might not have been recognized. The use of validated scales in the survey design increased the validity and reliability of the data. Finally, surveys allowed for the collection of holistic lifestyle data that explored physical, mental, and spiritual factors, providing us with a better perspective of social determinants that may have impacted the pandemic experience for this specific population.

### Study Limitations

Because this study was derived from a larger longitudinal study, there were limitations in terms of the sample size and the measurement of religiosity. First, our sample size for analyses was relatively small due to missing data at the second time point (our post hoc power analyses indicated that 84 participants had data collected at both time points) and limited the interpretation of some of the nonstatistically significant results that mostly, however, consistently approached significance.

This study was conducted among low-income women of mostly Mexican descent with low acculturation levels who were living in southern California. However, Latinas in other parts of the USA are far more diverse (from Puerto Rico, Columbia, El Salvador, Cuba or other countries) and may be more acculturated. Thus, one of our study limitations is the lack of generalizability to Latinas who are more acculturated and from other cultural backgrounds.

Although intrinsic religiosity and positive religious coping were examined in this study, negative coping was not. We also did not collect information on specific Protestant religions professed by Christian participants or on the intensity of religious practices. Finally, we did not control for factors that could also influence the outcomes, such as fear and stress. We nevertheless find our results compelling, as they provide a unique view of this issue during a time of high stress for this low-income population at high risk of exposure to COVID-19, as many either were working as “essential workers” or had a family member who was working as an “essential worker.”

## Conclusion

The findings from this study may be useful in designing and guiding low-dose interventions for persons most affected by a large-scale crisis similar to the COVID-19 pandemic. Awareness of these protective factors may be key in times of crisis, especially in the face of a shortage of culturally competent mental health professionals.

Pointing to the critical role of faith and a relationship with God (intentionalizing) could be part of outreach programs for persons in crises (Zoom church services, public messaging aimed at this subpopulation that is often ignored but is highly needed), in addition to facilitating and offering needed mental health services. Furthermore, encouraging religious coping behaviors within interventions addressing other health issues or even mental health issues might have a positive impact.

Future research should replicate this study with Latino men, among other Latinx subcultures, and with a larger sample size. More specific details on religious backgrounds and practices may also help to better assess how these factors affect the physical and mental health of these populations in times of crisis.

## Data Availability

The datasets generated during the current study are not publicly available, but de-identified data will be made available upon request where such requests are compliant with receipt of ethical approval from the sending and receiving hosts’ institutional ethics review boards.
